# Expansin genes expression in growing ovaries and grains of sunflower are tissue-specific and associate with final grain weight

**DOI:** 10.1186/s12870-018-1535-7

**Published:** 2018-12-04

**Authors:** Francisca M. Castillo, Javier Canales, Alejandro Claude, Daniel F. Calderini

**Affiliations:** 1 Graduate School, Faculty of Agricultural Sciences, Universidad Austral de Chile, Valdivia, Chile; 2 Plant Production and Plant Protection Institute, Faculty of Agricultural Sciences, Universidad Austral de Chile, Valdivia, Chile; 3 Institute of Biochemistry and Microbiology, Faculty of Sciences, Universidad Austral de Chile, Valdivia, Chile; 4 Millennium Institute for Integrative Biology (iBio), Santiago, Chile

**Keywords:** Expansin, Grain tissues, Grain weight, Source-sink ratio, Gene expression, Sunflower

## Abstract

**Background:**

Grain weight (GW) is a key component of sunflower yield and quality, but may be limited by maternal tissues. Cell growth is influenced by expansin proteins that loosen the plant cell wall. This study aimed to identify spatio-temporal expression of EXPN genes in sunflower reproductive organ tissues (ovary, pericarp, and embryo) and evaluate correlations between reproductive organ growth and expansin genes expression. Evaluations involved eight different developmental stages, two genotypes, two source-sink treatments and two experiments. The genotypes evaluated are contrasting in GW (Alybro and confection variety RHA280) under two source-sink treatments (control and shaded) to study the interactions between grain growth and expansin genes expression.

**Results:**

Ovaries and grains were sampled at pre- and post-anthesis, respectively. Final GW differed between genotypes and shading treatments. Shading treatment decreased final GW by 16.4 and 19.5% in RHA280 and Alybro, respectively. Relative expression of eight expansin genes were evaluated in grain tissues. EXPN4 was the most abundant expansin in the ovary tissue, while EXPN10 and EXPN7 act predominantly in ovary and pericarp tissues, and EXPN1 and EXPN15 in the embryo tissues.

**Conclusions:**

Specific expansin genes were expressed in ovary, pericarp and embryo in a tissue-specific manner. Differential expression among grain tissues was consistent between genotypes, source-sink treatments and experiments. The correlation analysis suggests that EXPN genes could be specifically involved in grain tissue extension, and their expression could be linked to grain size in sunflower.

**Electronic supplementary material:**

The online version of this article (10.1186/s12870-018-1535-7) contains supplementary material, which is available to authorized users.

## Background

In the last 50 years, oil crops such as soybean, sunflower, and oilseed rape have become increasingly important in international food trade, due to increased human consumption and demand of oil for biofuel production. Sunflower has had the third highest relative growth among crop commodities; it contributes to calorific intake [1] and provides about 8% of global oil production [2]. To meet the increasing food demand, sunflower grain and oil yield must both be improved. Grain weight (GW) is a key trait affecting sunflower yield and quality, yet a systematic understanding of physiological and molecular drivers of GW is still lacking for sunflower and other oil crops. Most studies of GW and grain size determination have focused on the grain filling period. Physiological studies have assessed associations between GW and key post-flowering factors such as maximum water content in wheat [3–5], maize [6, 7], and sunflower [8]. However, little is known about the genetic determination of grain water dynamics. Genetic studies have also focused on the post-flowering period, where GW has been identified as a quantitative trait controlled by multiple genes [9–13]. Scant information is available about the genetic control of GW during the pre-flowering phase, though it has recently been demonstrated that GW determination in sunflower is a continuous process from early pre-anthesis (R3 stage: reproductive stage when ovaries are growing) to physiological maturity (PM: when GW reaches its maximum value with a water content about 38%) [14], challenging the general assumption that flowering is a pivotal phenological stage for GW determination. In order to fully understand GW determination, an integrated physiological and molecular approach, linking the pre and post-anthesis periods, is necessary in sunflower.

The sunflower grain is an achene comprised of two main components: the pericarp (coat or hull), resulting from the fusion of the ovary tissues and part of the receptacle (maternal origin), and the embryo, formed by two cotyledons and a small stem and radicle, derived from the egg fertilization. The mature sunflower grain lacks endosperm (it is consumed during embryo growth), thus grain reserves (lipids, carbohydrates, and proteins) accumulate within embryo cells, mainly in the cotyledons [15].

The relationship between the pre- and post-anthesis periods has been attributed to the flower ovary, which becomes the pericarp after pollination in sunflower and other crops [4, 14, 16]. Grain maternal tissues (ovary/pericarp) undergo rapid cell division and expansion, which in turn may impose a physical limit to the endosperm or embryo of the grain, suggesting that maternal tissues control potential grain size [17–25]. However, these suggestions are still speculative and the physiological and molecular processes through which the continuous ovary-pericarp growth controls grain size are only starting to be known [3, 25–28].

Lindstrom et al., (2006) [29] and Castillo et al., (2017) [14], showed that GW of sunflower is much more sensitive than grain number to lower source-sink ratios during the period before flowering (R2 to R5), being R2 the stage immediately after floral initiation, when cell division in the ovary wall ceased, and R5 when anthesis of external flowers starts. In addition, Lindstrom et al., (2007) [30] showed that shading at pre-anthesis reduced GW and the number of pericarp middle layer strata, supporting the hypothesis that GW determination is a continuum process. Maternal tissues evolve from the ovary to the pericarp, a process driven by complex regulation involving cell division and expansion, utilization of assimilates, and the interaction of many genes and signals [31, 32]. Cell size depends on the cell capacity for enlargement and extension; while the plant cell wall must be strong enough to contain the turgor of plant cells, its extensibility also determines plant cell expansion [33, 34]. Several cell wall enzymes and proteins have been implicated in the loosening process that occurs during cell growth, including expansin (EXPN) proteins [35, 36] and xyloglucan endotransglucosylase/hydrolase [37]. EXPN have been known as “factors that loosen the cell wall”, playing a major role in plant cell growth by enabling plant cell expansion [38], among other processes ([39] and citations therein). However, the role of EXPN in grain growth is currently poorly understood.

In multigene families such as EXPN, different members may play unique developmental or tissue-specific roles, though there is currently little information about the role of EXPN in specific grain tissues. Lizana et al. (2010) [3] conducted an experiment on wheat and found clear relationships between grain size dynamics, water content, and EXPN expression in pericarp tissues. Meanwhile, when overexpressed in Arabidopsis, the sweet potato EXPN1 gene (IbEXP1) positively affected grain size and brassinosteroid signaling pathways [40]. In barley, the gene expression of nine genes involved in cell wall biosynthesis shows a broad maximum between 3 and 10 days after flowering [41]. Much less information is available about the role of EXPN in sunflower grain growth, thus this study aimed to address the following questions: i) are ovary and grain growth associated with EXPN expression at both pre- and post- flower fertilization? ii) is pericarp and embryo enlargement driven by different EXPN? iii) is the timing of EXPN expression similar for the pericarp and embryo? and iv) is the effect of EXPN on GW mediated by the abundance, rate, or duration of EXPN expression? To answer these questions, we identified the EXPN involved in ovary and grain growth by measuring mRNA gene transcripts and their time-course expression in field experiments. A high-quality reference database for the sunflower genome (3.6 gigabases) was also utilized, together with extensive transcriptomic data from vegetative and floral organs [42].

## Results

###  Environmental conditions, crop phenology, and grain weight

Climate conditions were similar between experiments. Mean temperature during the Emergence-R1 period was only 1.1 °C higher in experiment 1, while the R1 to PM average temperature was higher in experiment 2, primarily during R2-R5, when the mean temperature was 3 °C higher in experiment 2 than in experiment 1 (see Table A1 in [14]). Crop development was similar in both growing seasons in Alybro reaching anthesis (R5) at 74 days after emergence. In experiment 2, phenology between Alybro and RHA280 was similar, the R5 and PM was later in Alybro than in RHA280 but only by 2 days at each stage (see Fig. 1 in [14]).

**Fig. 1:**
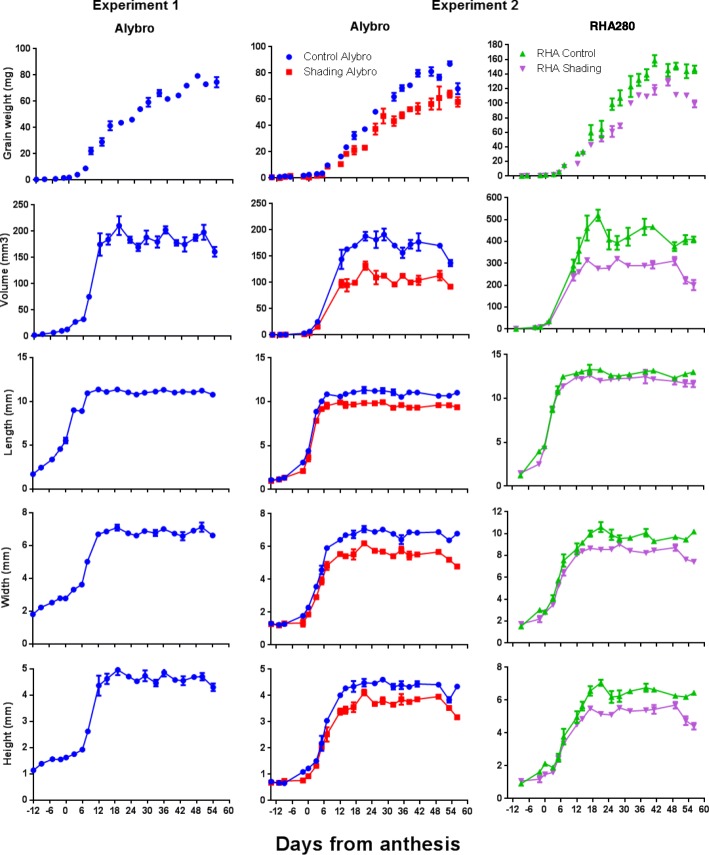
Grain growth dynamics of two sunflower genotypes under source-sink treatments. **a**. Grain weight. **b**. Grain volume. **c**. Grain length. **d**. Grain width. **e**. Grain height dynamics. Control and shading treatments are shown for experiment 2: Blue = Alybro control; Red = Alybro shade; Green = RHA280 control; Purple = RHA280 shade

Final GW of Alybro was 76.5 mg in experiment 1 and 79.2 and 58.2 mg in experiment 2 under control and shaded treatments, respectively. As expected, the confection genotype RHA280 reached higher (*p* < 0.05) GW than Alybro in both the control treatment (148.8 mg, 47% higher) and under shading (107 mg, 46% higher) of experiment 2 (Table 1). Ovary weight of Alybro at R3 and R5 was slightly higher in experiment 1 than in experiment 2 (Table 1). In the last experiment, RHA280 ovary weight was significantly greater (p < 0.05) than the ovary weight of Alybro at both R3 and R5 phenological stages [14]. The reduction of the source-sink ratio by the shading treatment had a strong impact on ovary weight at anthesis (*p* < 0.001), decreasing it by 45 and 33% in Alybro and RHA280, respectively (Table 1). The shading treatment also negatively affected grain dimensions (*p* < 0.001); grain length was reduced by 13 and 5.4% in Alybro and RHA280, respectively, while grain width decreased by 19% in Alybro and 14% in RHA280, and grain height decreased by 16 and 20% in Alybro and RHA280, respectively (Table 1).

**Table 1:** Physiological traits of grains in experiments 1 and 2 of sunflower genotypes

Exp	Genotype	Source-Sink treament	Dry weight (mg)						Dimensions (mm)			
			Ovary at R3	Ovary at R5	Grain	Pericarp	Embryo	Grain/pericarp	Length	Width	Height	Embryo length
1	Alybro	Control	0.33	1.94	76.5	20.3	58.0	3.9	11.1	6.8	4.6	8.7
2	Alybro	Control	0.24	1.60	79.2	20.2	60.9	3.9	11.0	6.8	4.4	8.4
		Shading	0.23	0.88	58.2	14.1	46.4	4.1	9.6	5.5	3.7	7.5
	RHA280	Control	0.41	2.60	148.8	76.1	73.2	2.0	13.0	9.8	6.5	9.9
		Shading	0.37	1.75	107.0	58.1	61.5	1.8	12.3	8.4	5.2	9.0
	s.e.m		0.03	0.23	10.4	6.6	6.2	0.4	0.4	0.5	0.3	0.3
	Genotype		^a^	^a^	^a^	^a^	^a^	^a^	^a^	^a^	^a^	^a^
	Source-sink ratio		ns	^a^	^a^	^a^	^a^	*	^a^	^a^	^a^	^a^
	Genotype x Source-sink		ns	ns	ns	^a^	^a^	ns	^a^	ns	^a^	^a^

Values are means of three replicates. *ns* means not significant effects. * Significant effects at *P* < 0.05. ** Significant effects at *P* < 0.01

^a^Significant effects at *P* < 0.001 (modified from Castillo et al., 2017)

Figure 1 illustrates the time-course of GW, grain volume and dimensions across experiments 1 and 2. Alybro showed similar GW, volume, and dimension dynamics in both experiments, while RH280, evaluated only in experiment 2, reached higher rates than Alybro across all measured traits (Fig. 1). The lower source-sink ratio before anthesis (under the shading treatment) reduced GW and volume in both Alybro and RHA280 compared to the control treatment, mainly by decreasing the grain filling rate (Fig. 1).

**Fig. 2:**
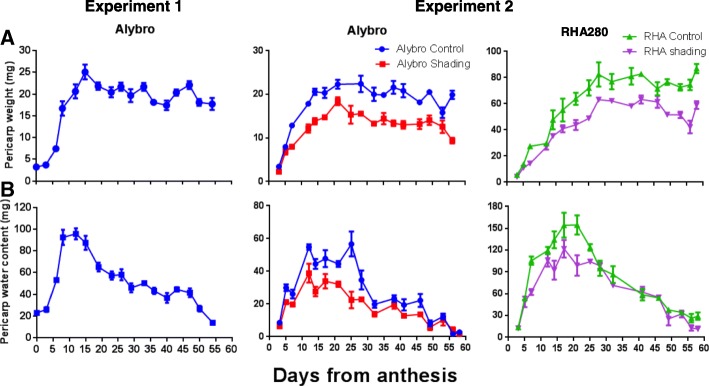
Pericarp growth dynamics of two sunflower genotypes. **a**. Pericarp weight. **b**. Pericarp water content. Control and shading treatments are shown for experiment 2: Blue = Alybro control; Red = Alybro shade; Green = RHA280 control; Purple = RHA280 shade

In experiment 2, the differences between the control and shading treatments were observed early during grain growth (+ 6 days from anthesis, DFA) in both pericarp weight and water content (Fig. 2). Pericarp weight dynamics were similar between the two growing seasons for Alybro (Fig. 2 a), though the maximum pericarp water content was higher in experiment 1 than in experiment 2 (Fig. 2b).

### Classifying sunflower EXPN by phylogenetic relationships and comparative analysis of the gene structure

**Fig. 3:**
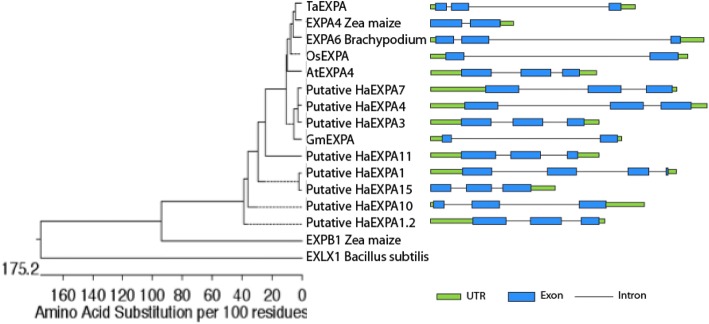
Phylogenetic tree of eight putative EXPN of sunflower, orthologs of EXPN7, EXPB1, EXLX1. Phylogenetic relationship of Arabidopsis, soybean, rice, maize, wheat, brachypodium, and sunflower EXPN genes. The phylogenetic tree was constructed based on a conserved domain of predicted protein sequences using MegAlign software. The gene model is indicated to the right of each EXPN

Genome databases were used to evaluate the role of EXPN in specific tissues of sunflower grains. According to the phylogenetic tree, the sunflower EXPN selected in this study are part of the α-EXPN subfamily, and the gene models showed that each EXPN had a conserved intron/exon structure and protein domain, supporting their close evolutionary relationship (Fig. 3). In the α-EXPN subfamily, all sunflower EXPN genes had 3 exons and 2 introns, and orthologs (soybean, rice, and maize) had 2 exons and 1 intron (Fig. 3). Sunflower EXPN proteins share 65.9–95.9% of their identity with each other, and over 60% of their identity is shared with EXPN7 orthologs (Additional file 1: Figure S1). As expected, the identity shared among EXPN from different groups was low: maize β-EXPN (EXPNB1) shared 26.8–29.1% identity with α-EXPN, while EXLX1 shared 14.9–18.9% identity with α-EXPN (Additional file 1: Figure S1).

### EXPN expression is arranged in a temporal and tissue-specific manner during ovary and grain growth

**Fig. 4:**
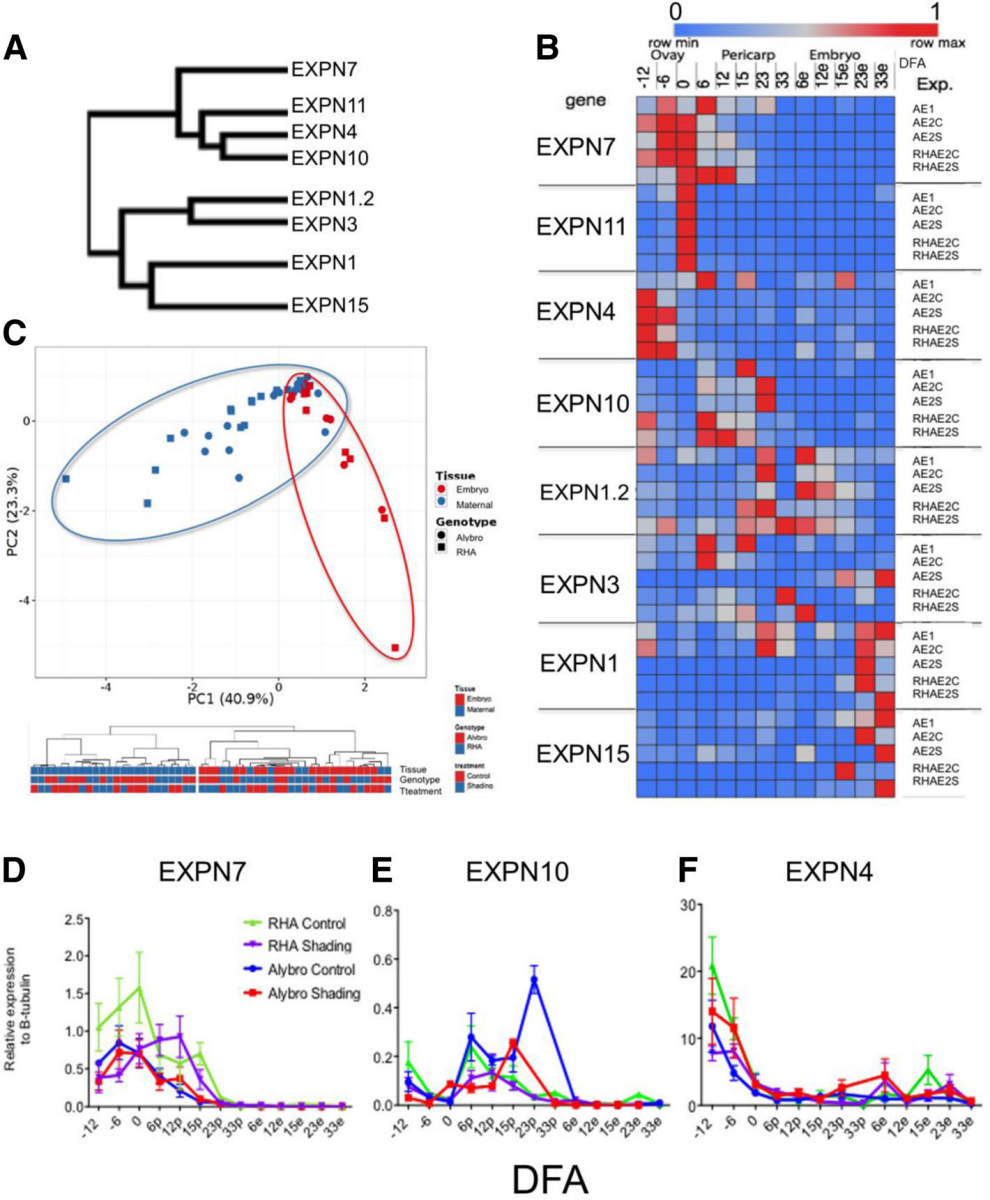
Clustering, heatmap and principal component analysis (PCA) of EXPN expression in sunflower ovaries and grains (pericarp and embryo). **a**. Clustering based on EXPN expression patterns. **b**. Heatmap showing EXPN expression patterns. Expansin expression levels were compared by Z score using the Clustvis bioinformatics tool. Columns names indicate the days from anthesis (DFA) in which the expression was measured (e = embryo). Rows name on the left show the EXPN genes evaluated and row names on the right indicate the experiment where EXPN expression was evaluated, including genotype, treatment, and growing season (A = Alybro; RH = RHA280; E1 = experiment 1; C = control; S = shade). **c**. PCA with all EXPN expression data grouped into two main groups according to tissue expression (blue and red circle in PCA). **d**. Relative expression of EXPN7 to β-tubulin. **e**. Relative expression of EXPN10. **f**. Relative expression of EXPN4

The “Clustvis bioinformatics tool” [42] was used to visualize the gene expression patterns of the eight selected EXPN genes and to perform a comparative gene expression analysis along the grain growth across genotypes, experiments and treatments. EXPN gene clustering between both experiments indicated two main groups, according to their expression patterns (Fig. 4a). In one group, EXPN were expressed mainly in maternal tissues (EXPN7, EXPN11, EXPN10, EXPN4), while in the other they were mainly expressed in embryo tissues (EXPN1, EXPN1.2, EXPN3, EXPN15). The clustering and heatmap showed that two EXPN from each group have similar expression patterns across experiments and genotypes, i.e. EXPN 7 and 11 in pericarp, and EXPN 1 and 15 in embryo tissues (Fig. 4). Differential expression among grain tissues was consistent between genotypes (Fig. 4b). PCA was performed with relative gene expression of EXPN7, EXPN11, and EXPN4 (mainly expressed in maternal tissues) and EXPN1 and EXPN 15 (predominantly expressed in embryo tissues), accounting for 64.2% of the total variation (Fig. 4c). When PCA of the relative EXPN gene expression was performed using the entire dataset, a lower percentage of the total variation (45.2%) was found (data not shown). Expression data was grouped into two main groups according to tissue expression (Fig. 4c). EXPN4 showed more abundant relative expression (mainly in the ovaries; Fig. 4f) compared to all other EXPN evaluated in this study, followed by EXPN1 and EXPN7 (Additional file 2: Figures S2L and 4D, respectively).

EXPN11 had the most consistent expression pattern across genotypes, treatments, and experiments, peaking at anthesis in both experiments, 1 and 2 (Fig. 4b). The relative abundance of EXPN11 was higher in RHA280 than in Alybro in experiment 2, but the shading had little effect on relative abundance (Additional file 2: Figure S2J). EXPN7 was expressed in maternal tissues of both genotypes, though in experiment 1, it peaked at + 6 DFA in the pericarp of Alybro, whereas under the control treatment of experiment 2, it peaked at anthesis in the pericarp of both Alybro and RHA280, before decreasing. In both experiments, EXPN7 was expressed earlier in ovary tissues, from − 12 DFA (Fig. 4b, d). In experiment 1, EXPN4 was expressed in the ovary of Alybro from − 12 DFA, peaking at + 6 DFA in the pericarp. In experiment 2, EXPN4 was more consistent in its expression patterns between the two genotypes; it was more abundant in ovary tissues (peaking at − 12 DFA) under control treatments, and under shading treatments maintained high expression levels between − 12 and − 6 DFA in both genotypes. EXPN4 was also expressed in embryo tissues in both genotypes and experiments, but at lower relative expression levels (Fig. 4b, f). In Alybro (both experiments) EXPN10 expression in the pericarp peaked between + 15 and + 23 DFA, whereas the peak in RHA280 was earlier, at + 6 and + 12 DFA (Fig. 4b,e).

EXPN1.2 was expressed in both pericarp and embryo tissues. Expression was higher in the pericarp, which peaked later than other EXPN in the pericarp (+ 23 DFA in Alybro for both experiments, and between + 15 and + 23 DFA in RHA280). On the other hand, the peak expression of EXPN1.2 in the embryo was at + 6 DFA in control treatments of both genotypes (Fig. 4b). The shading treatment decreased the expression levels of EXPN1.2, mainly in the pericarp, and shifted the RHA280 peak from + 23 to + 33 DFA (Fig. 4b; Additional file 2: Figure S2K). Meanwhile EXPN1 was expressed mainly in embryo tissues and later than other EXPNs (+ 23 and + 33 DFA) in both genotypes and experiments. Similarly, mRNA of EXPN15 was detected preferentially in the embryo at + 15, + 23 and + 33 DFA in both genotypes and experiments (Fig. 4b).

### Correlation between physiological traits of grain and EXPN expression patterns in sunflower

To explore if physiological grain traits correlate with EXPN expression patterns, we performed a correlation analysis using all data from two experiments. We used the Gini correlation coefficient to estimate the relationship between phenotypic traits and gene expression levels. The Gini correlation coefficient can compute the correlation of two variables considering both rank and value information. For this reason, this methodology is more robust on non-normally distributed data and is more stable for data containing outliers than the widely used Pearson correlation coefficient.

Qualitatively, EXPN7 follows a similar – but inverse – time course than the grain growth pattern (Additional file 2: Figure S2G). Specifics EXPNs associated with the extension of the ovary, pericarp and embryo. EXPN7, EXPN10, and EXPN11 were found to be specific to maternal tissues (Fig. 4b, Additional file 2: Figure S2), while EXPN1 and EXPN15 were more abundant in embryo tissues (Fig. 4b, Additional file 2: Figure S2).

**Fig. 5:**
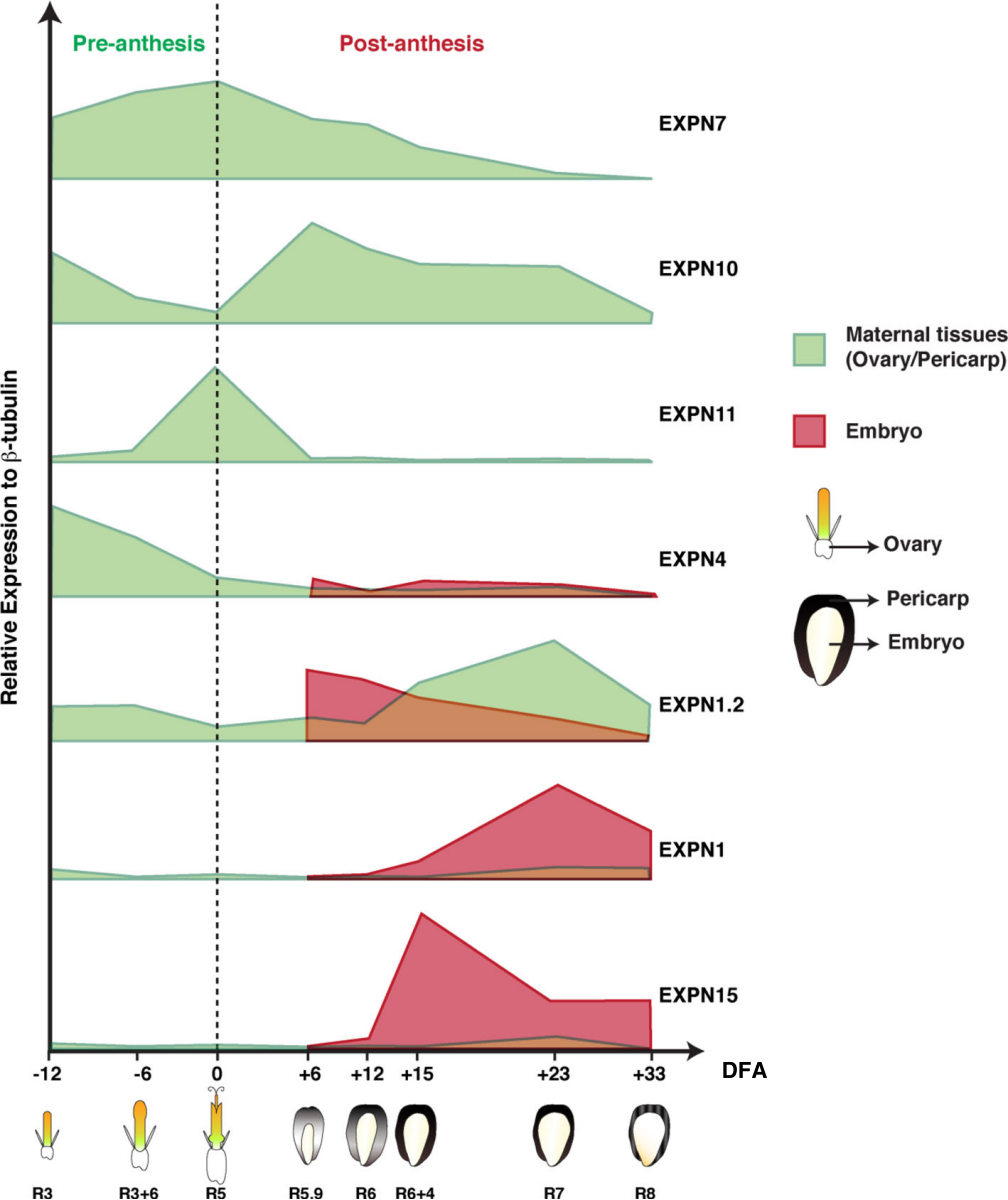
Schematic model representing expression patterns of seven EXPN genes during sunflower ovary and grain growth. Relative expression of EXPN genes is based on qRT-PCR analysis from samples harvested at various days from anthesis. The development stage according to Schneiter and Miller (1981) scale is indicated below each ovary and grain. The dashed line divides pre- and post-anthesis periods (DFA = Days from anthesis)

Physiological traits that showed significant correlations with EXPN expression patterns over time (at eight moments of sunflower development, Figs. 4, 5) are listed in Tables 2 and 3. Negative correlations mean that the grain growth dynamics follow a similar but inverse time course to the EXPN expression patterns, i.e. EXPN transcripts were accumulated when grain growth rates started and expression decreased according to the growth of the ovary/grain. The highest negative correlation was found between physiological traits and EXPN10 patterns in the maternal tissues, where the Gini correlation coefficient ranged from − 0.70 to − 0.86 (Table 2). GW was negatively correlated with EXPN7 (− 0.72), and most of the assessed physiological traits negatively correlated with the relative expression of EXPN7 in both the ovary and pericarp, ranging from − 0.50 to 0.72 (Table 2).

**Table 2:** Correlation between physiological trait dynamics and EXPN expression patterns in maternal tissues. Gini correlation coefficient and corresponding levels of significance between physiological trait dynamics and EXPN expression patterns in maternal tissues (ovary and pericarp)

Physiological traits dynamics	EXPNs expression patterns in maternal tissues	Correlation coefficient	*P*-value
Grain volume	EXPN10	−0.84	0.000
Grain volume	EXPN7	−0.52	0.002
Grain weight	EXPN7	−0.72	0.000
Grain weight	EXPN10	−0.83	0.000
Pericarp weight	EXPN10	−0.71	0.000
Pericarp weight	EXPN7	−0.58	0.000
Pericarp weight	EXPN4	0.42	0.023
Embryo weight	EXPN7	−0.68	0.000
Embryo weight	EXPN10	−0.72	0.000
Water content	EXPN10	−0.85	0.000
Length	EXPN10	−0.86	0.000
Length	EXPN7	−0.50	0.002
Width	EXPN10	−0.81	0.000
Width	EXPN7	−0.53	0.001
Height	EXPN7	−0.59	0.000
Height	EXPN10	−0.80	0.000
Embryo length	EXPN10	−0.70	0.000
Embryo length	EXPN7	−0.54	0.000

**Table 3:** Correlation between physiological trait dynamics and EXPN expression patterns in embryo tissues

Physiological traits dynamics	EXPNs expression patterns in embryo tissue	Correlation index	*P*-value
Grain volume	EXPN15	0.74	0.000
Grain volume	EXPN1	0.56	0.002
Grain volume	EXPN1.2	−0.58	0.004
Grain weight	EXPN4	−0.76	0.000
Grain weight	EXPN1	0.73	0.000
Grain weight	EXPN15	0.77	0.000
Pericarp weight	EXPN15	0.74	0.000
Embryo weight	EXPN4	−0.71	0.000
Embryo weight	EXPN1	0.75	0.000
Embryo weight	EXPN15	0.63	0.002
Length	EXPN1.2	−0.66	0.000
Length	EXPN15	0.75	0.000
Width	EXPN15	0.71	0.000
Width	EXPN1.2	−0.61	0.001
Width	EXPN1	0.53	0.010
Height	EXPN15	0.75	0.000
Height	EXPN1	0.67	0.001
Height	EXPN4	−0.48	0.020
Embryo length	EXPN15	0.74	0.001

Gini correlation coefficients and corresponding levels of significance between physiological trait dynamics and EXPN expression patterns in embryo tissue

In embryo tissues the best correlations were shown by EXPN4 and EXPN1, i.e. -0.76 between grain weight and EXPN4 and 0.75 between embryo weight and EXPN1 (Table 3). EXPN1 had significant positive correlations with grain volume, GW, embryo weight, grain width and grain height, while EXPN1.2 was associated with grain volume, grain length, and grain height (Table 3). EXPN15 correlated positively with all the physiological grain traits evaluated in this study (correlation coefficient of 0.63–0.77).

The expression patterns of individual EXPN were consistent between both seasons for most of the EXPN genes evaluated in this study, except for EXPN3 (Fig. 4b). A schematic model summarizing the most consistent data recorded in both experiments is shown in Fig. 5.

## Discussion

This study aimed to: i) identify EXPN genes expressed in sunflower reproductive organs (e.g. floret ovaries and grains; ii) assess tissue-specific EXPN expression in those organs; and iii) evaluate correlations between reproductive organs and EXPN expression. We identified eight putative EXPN acting in reproductive tissues (ovary, pericarp, and embryo) during the pre- and post-anthesis periods. Evaluations involved eight different developmental stages, two genotypes, and two source-sink treatments in two experiments. We found that all the EXPN selected using in silico analysis and the transcriptomic data, share a similar protein-conserved domain and a relatively simple intron/exon structure belonging to the α-EXPNs subfamily (Fig. 1).

Expression pattern analysis using qRT-PCR supports the hypothesis that EXPN genes act in a tissue-specific and temporal manner in sunflower grains. Taking into account that other sunflower organs and tissues were not evaluated in this study, we only highlight its expression in reproductive tissues from the pre-flowering to post-flowering stages, considering that they may be acting in other organs. Similarities in expression patterns could indicate functional redundancy between EXPN of sunflower reproductive tissues, as was previously reported in grasses and other plant groups [43, 44]. Organ specific expression of Os-EXP1, Os-EXP2, and Os-EXP4 was found in rice [45]. A study of maize reproductive organs found at least 21 ZmEXPNs genes, 16 of which were predominantly expressed in the tassel, while five ZmEXPNs were mainly expressed in the endosperm, suggesting their involvement in endosperm development and growth [46].

A central question of the present study was if differences in GW between genotypes and shading treatments were explained by the abundance or duration of EXPNs expression. The difference between Alybro and RHA280 genotypes (with different GW) was ascribed to relative abundance. In agreement with this response, most of the EXPN genes of both genotypes showed lower abundance under the lower source treatment (Fig. 4, Additional file 2: Figure S2). In addition, the negative effect of the source reduction on GW could be mediated by a later timing of the peak of EXPN expression. These finding agree with the decrease of the ovary growth rate by the shading treatment and a lower grain and pericarp growth rate reported previously of the evaluated genotypes [14]. A recent study showed that the transcript abundance of genes involved in cell expansion, such as EXPN genes, were significantly higher in the large-seeded chickpea cultivar [47], supporting our results of the different relative abundance between sunflower genotypes.

Cell expansion determines organ size and is powered by water uptake and expansion of the cell wall [48]. To understand the potential roles of grain water uptake and loss during grain filling the timing of key grain growth events is necessary. Grain water content serves as an engine to increase the turgor pressure in the vacuole powering cell expansion. Therefore, once grain desiccation commences, the driving force disappears and grain enlargement ends (at this time, all grain dimensions reach their maximum values) and this timing agrees with the expression pattern of EXPN7 and EXPN10. The time course of EXPNs expression was shown tissue-specific and seems to control the enlargement of the ovary, pericarp and embryo. The expression of EXPN4, EXPN7, EXPN10 and EXPN11 were found mainly in maternal tissues. Among them, EXPN4 was more abundant in the ovary/pericarp. On the other hand, EXPN1 (embryo), EXPN1.2 and EXPN 10 (pericarp) were expressed late during grain filling (between + 15 and + 33 DFA) in sunflower grains. These EXPNs could play a role in grain ripening, as it was previously reported in other crops, linking the EXPNs with grain or fruit maturation [49, 50].

Previous studies have shown expression analysis of genes related to cell wall and cell expansion. H + -ATPases acidify cell walls, which activates EXPN, leading to cell wall synthesis and cell expansion [34]. Radchuk et al. (2011) [41] reported gene expression profiles of EXPN, together with the expression of related genes such as H + -ATPases, and enzymes of cell wall biosynthesis from the microarray data set in barley pericarp. In their study, five EXPN genes reached the highest expression 3–4 DFA and H + -ATPase exhibited the highest gene expression 2–10 DFA. The maximum expression of nine genes involved in cell wall biosynthesis was 3–10 DFA. This pattern of gene expression aligns with pericarp growth, where cell expansion and cell wall synthesis occur 3–10 DFA in barley [41]. Our study of sunflower found that EXPN7, EXPN 4, EXPN10, and EXPN11 would play key roles early in grain development, until pericarp growth levels off soon after flowering, i.e. + 8 DFA at R5.1. Maximum pericarp water content was attained by + 10 DFA (Fig. 3), concurring with other studies [8, 51] and reinforcing the link between EXPN expression and water dynamics of sunflower grains. In our study, EXPN expression patterns suggest an earlier specific role (from − 12 to + 6 DFA) for EXPN7 and EXPN10 in the ovary and pericarp, respectively, compared with other isoforms. This agrees with Lindström and Hernández (2015) [51], who demonstrated that final pericarp size is attained at + 8 DFA when secondary wall deposition in the pericarp cells begins.

The correlation analysis supports the qualitative analysis (heatmap, expression dynamics, and grain growth dynamics) and suggests that EXPN genes could be specifically involved in grain tissue extension, and their expression could be linked to grain size in sunflower (Fig. 5). EXPN4 was abundant in ovary tissues, while EXPN10 and EXPN7 were specifically expressed in the ovary and pericarp tissues, and EXPN1 and EXPN15 in the embryo. These results would indicate that EXPN isoforms are linked to flower and grain growth in sunflower.

On the other hand, the grain growth process integrates and coordinates different pathways like genetics, epigenetics, metabolic, physiological, and environmental factors [52–57]. Several genes acting in maternal tissues have been identified in different plant species [19, 25, 58–62]. For example, introgression of the mutant TaGW2-A1 allele (a gene that negatively regulates cell number in maternal tissues) showed an association between final GW and carpel size in wheat [63], reinforcing the importance of maternal tissues on GW determination. Furthermore, recent studies found that pericarp cell length correlates with, and affects the, final grain size and weight in wheat and tomato [25, 62, 64]. Meanwhile, transgenic overexpression of GhRDL1 (a cell wall protein that interacts with GhEXPN1) in cotton and GhEXPN1 in Arabidopsis produced more and larger grains in both species [40, 65]. Moreover, the overexpression of sweet potato EXPN gene IbEXP1 in Arabidopsis, under the control of the 35S promoter, also resulted in larger grains than the control plants [40]. Previous studies have been shown, through overexpression and/or RNAi approaches, that EXPN proteins are a key for fruit ripening, growth of root hairs, tolerance to abiotic and biotic stresses, among others [39]. Our findings of specific EXPN (e.g. EXPN4, EXPN7, and EXPN10) expressed in maternal tissues of sunflower grains enable us to hypothesize that these EXPN are a key component of ovary/pericarp growth. The present findings and the previous knowledge about the involvement of EXPNs on grain growth of crops [3, 21, 39, 41, 65], allow us to speculate that results of this study provide tools for improving sunflower GW by cloning and/or overexpressing them as it was shown in the model plant Arabidopsis where grain size was increased [40] and improved grain production in Tobacco [66]. Alternatively, the development of molecular markers based on information reported in the present study could also be useful for breeding programs.

## Conclusions

The molecular and physiological bases of GW and grain size determination can be studied in an integrated manner by using a quantitative molecular approach combined with physiological and agronomical studies. Using qualitative and quantitative analysis of grain growth and expression dynamics, combined with heatmap and correlation analysis, we identified eight putative EXPN genes that could be involved in grain tissue extension. EXPN4 was the most abundant EXPN in the ovary tissues, while EXPN10 and EXPN7 act predominantly in ovary and pericarp tissues, and EXPN1 and EXPN15 in the embryo tissues. These results suggest that EXPN genes may control grain growth in sunflower from the early phases of development. Interestingly, EXPN7 and EXPN10 gene expression in the pericarp leveled off soon after flowering (+ 8 DFA), which is close to the maximum pericarp water content reached at + 10 DFA, indicating that EXPN and maternal tissue water dynamics may be linked in sunflower. These results contribute to improve the understanding of GW and grain size determination in sunflower and other grain crops.

## Methods

### Experiments, treatments, and field conditions

Two field experiments were carried out at the Experimental Station of the Universidad Austral de Chile in Valdivia (39°47’S, 73°14’W) during the 2013/14 (experiment 1) and 2014/15 (experiment 2) growing seasons. Experiments were designed to evaluate relationships between EXPN expression patterns and sunflower reproductive organ dynamics (ovary weight, GW, and grain water content and dimensions), both at pre- and post-flowering.

In experiment 1, plant reproductive organs and EXPN expression were measured in the sunflower oilseed hybrid Alybro, which corresponds to a hybrid with a short cycle and adapted to the environmental conditions of southern Chile. It was sown on November 2 in 2013 in a randomized complete design with three replicates. Experiment 2, aimed to validate the results of the first experiment by including an additional sunflower genotype contrasting in GW (confection variety RHA280, also with a short cycle and similar phenology to Alybro), and two source-sink treatments (control and reduced source-sink ratio prior to anthesis), with the aim of decrease GW, and add a new scenario to study determinants of GW. This experiment was sown on 1 November 2014 in a split plot design, where main plots were assigned to source-sink treatments and sub-plots to the genotypes, with three replicates. Source-sink treatments were established by shading the plots from stages R2 to R5, for 16–17 days (see Table A1 in [14]). Shading treatments comprised black nets that intercepted 80% of incident radiation. Nets were supported by wooden structures over the treated plots as in previous evaluations [29, 67] and reported by Castillo et al. (2017) [14].

In both experiments, seeds of Alybro genotype were provided by Panam Chile and seeds of the confection variety RHA280 by Dr. Laura Marek from USDA-ARS, NCRPIS. Plots were 5 m long with 10 rows, 0.70 m apart and a seeding rate of 6 plants m^− 2^. Each plot was fertilized at sowing with 100 kg P_2_O_5_ ha^− 1^ and 132 kg K_2_O ha^− 1^. Nitrogen fertilization was applied using 150 kg N ha^− 1^ at sowing and 150 kg N ha^− 1^ when floral stems appeared. Weeds, insects, and diseases were controlled in both experiments and drip irrigation was applied to avoid water stress.

### Phenology and plant sampling

Crop phenology was recorded twice a week during the crop cycle (in both experiments), according to the scale proposed by Schneiter and Miller (1981) [68]. Flowers or grains were sampled from R3 to maturity every three days. Two capitula per replicate and peripheral grains (florets in the 3–9 circles counted from the outside of the head) were harvested from each sample. Fresh and dry matter, and flower or grain dimensions (divided into pericarp and embryo), were measured. Water content of flower, pericarp, and embryo tissues was calculated as the difference between fresh and dry weight. Grain dimensions (length, width, and height) were recorded quickly after being sampled in a subset of four peripheral grains per capitulum, using an electronic caliper (digital caliper, China) as in Hasan et al. (2011) [4]. At harvest, five capitula were sampled per repetition to measure average GW (from 0.25 of capitulum) in both experiments.

Grain volume was measured by water displacement of 10 grains per experimental unit at maturity. This procedure mirrors that used in other studies for wheat [69], sorghum [20, 70], maize [71], and sunflower [8]. For molecular analysis, another set of harvested flowers and grains were immediately preserved in cryotubes and immersed in liquid nitrogen. Each sample consisted of 10 ovaries (pre-anthesis sampling) and five grains (post-anthesis sampling) per capitulum, and were stored at − 80 °C until processing for gene expression evaluation.

### Molecular analysis: In silico analysis and primer design

Identification of putative EXPN genes expressed in sunflower grain tissues.

The first objective was to identify in silico EXPN genes expressed in sunflower reproductive organs. Well-characterized EXPN from *Zinnia elegans* (a relative of sunflower) were used as a query sequence (e.g. ZeEXP3: GenBank: AF230333.1) to search public databases of sunflower genome and transcriptomic data (https://www.heliagene.org) using the BLAST tool. Putative EXPN genes were identified based on gene annotation, bioinformatics, and RNA sequencing analyses. In the sunflower genome portal, protein-coding genes were annotated using a three-step process considering reciprocal best hits in the SwissProt and TAIR10 databases (12,360 sunflower proteins), protein-domain content in Interpro (26,646 sunflower proteins), and similarity with plant proteomes (Ensembl release 30) or coverage of the transcript with RNA-sequencing data [42].

From the BLASTp results, we chose eight putative EXPN according to the expression patterns tool in the sunflower transcriptome database (Additional file 3: Figure S3). We selected EXPN that were expressed in grains, whether expressed specifically in grains as EXPN15 (Additional file 3: Figure S3 A) or expressed in grains and other tissues such as leaves, roots, style, ligule, stem, stamen, corolla, and bract (e.g. EXPN4, Additional file 3: Figure S3 B). Then we selected EXPN sequences expressed mainly in grains, with high expression levels (reads per kilobase per million mapped reads). We also performed BLASTp in the Heliagene platform to find orthologous genes of each sunflower EXPN in Arabidopsis, Brachypodium, and soybean proteome from predicted proteins (Table S1). All sunflower EXPN explored in grain tissues identified highly with their orthologs (most > 70%), indicating that the protein domain is highly conserved in plants. Additional file 4: Table S1 shows the physical locations of EXPN on the sunflower genome, with each EXPN gene located on a different chromosome. Open reading frame length ranged from 1473 bp (EXPN15) to 3239 bp (EXPN4), with an average of 2368 bp. The identified EXPN genes encoded proteins ranging from 254 (EXPN11) to 311 (EXPN1.2) amino acids in length, with an average of 269 amino acids (Additional file 4: Table S1).

The eight sunflower EXPN (and the EXPN7 ortholog) were subjected to multiple sequence alignments using the MegAlign program with the CLUSTAL W algorithm [72]. The alignment between EXPN of sunflower and other species confirms the presence of two conserved domains in sunflower EXPN (Additional file 5: Figure S4). Selected sequences were also aligned to reveal the number of unique sequences. Sequences were searched against the non-redundant GenBank DNA and protein database using BLASTn and BLASTx [73, 74] and against the Uni Prot database resources using BLASTx. Sequences were used in BLASTx searches to confirm that they correspond to EXPN transcripts. Nucleotide sequences were also translated into protein using the ExPASy bioinformatic tool (https://web.expasy.org/translate/) to mark off the coding region for designing specific primers.

### Primer design

Specific primers were designed using the “PRIMIQUE” tool to detect different sequences of the gene family [75]. Two primer pairs were chosen for the same sequence, then tested and standardized for qPCR. We conducted a bibliographic search for sunflower reference genes that could be used as an endogenous control to normalize the data for differences in input RNA and the efficiency of reverse transcription between the various samples. We evaluated primers reported by previous studies for sunflower grain elongation factor 1 (EF1), S19 protein, β-tubulin, actin, ubiquitin, and 18S [73–80].

### RNA extraction and RT-PCR

Total RNA was isolated using the RNeasy Plant Mini kit (Qiagen), according to the manufacturer’s instructions. The kit provides a choice of lysis buffers depending on the amount and type of secondary metabolites in the tissue, thus standardizing the RNA extraction protocol. RNA quality and concentration was measured using spectroscopy with NanoQuant (Infinite M200, TECAN).

The isolated RNA was pretreated with DNaseI. First-strand cDNA was synthesized from 250 ng RNA using the ImProm-II™ Reverse Transcription System. The oligo(dt)16–18 primer/template mix was thermally denatured at 70 °C for 5 min and chilled on ice. A reverse transcription reaction mix was assembled on ice and contained nuclease-free water, reaction buffer, reverse transcriptase, magnesium chloride, dNTPs, and ribonuclease inhibitor. We added 1 U/μl of Recombinant RNasin® Ribonuclease Inhibitor before the template-primer combination was added to the reaction mix on ice. Following an initial annealing at 25 °C for 5 min, the reaction was incubated at 42 °C for up to 1 h. The synthesized cDNA (20 μl) was stored at − 20 °C. As a negative control, an RNA sample was replaced by water in this procedure.

### Quantifying EXPN mRNA levels using real time PCR (qPCR)

The qPCR reaction was performed in a final volume of 25 μL, containing 12.5 μL Brilliant II SYBR Green PCR Master Mix (Stratagene, Agilent technologies), 1 μL 10 μM forward and reverse primers and 8.5 μL of sterile deionized water. After an initial DNA polymerase activation step at 95 °C for 10 min, samples were subjected to 35 amplification cycles (95 °C for 15 s, 60 °C for 15 s, 72 °C for 15 s). No-template and no-transcriptase controls were included to detect genomic DNA contamination.

A melting curve was generated by incubating the reaction at 95 °C for 15 s, 25 °C for 1 s, and 70 °C for 15 s, and then slowly increasing the temperature to 95 °C. The mRNA abundance of EXPN genes between grain tissues and development stage was determined using the method proposed by Pfaffl (2001) [81], where β-tubulin was used as an internal control. Gene expression files were exported and uploaded into LinRegPCR software for quantification analysis [82]. The normalization factor was calculated as the geometrical mean of the RT-qPCR data obtained from LinRegPCR analysis. The underlying mathematical algorithm calculates qPCR efficiencies via linear regression in the exponential part of the fluorescence curve [82]. After confirming the amplified specific products, a standard curve of each primer pair was created with the amplification product. A dilution of 1:1000 was prepared before seven serial dilutions were prepared by a factor of 10, starting from the 1:1000 dilution of the previously amplified product. This was used to score the efficiency of the primers.

EXPN sequences were further verified by sequencing and the resulting chromatograms were viewed, evaluated, and aligned. Gene sequences were subjected to a homology search in the Heliagene portal (https://www.heliagene.org/) and National Center for Biotechnology Information database (https://blast.ncbi.nlm.nih.gov/Blast.cgi). To classify sunflower EXPN proteins into subfamilies, we searched orthologs of EXPN7 in Arabidopsis, soybean, rice, wheat, Bachypodium, and maize, and incorporated two non-related EXPNs such as a β-EXPN EXPB1 from maize and EXLX1 from Bacillus subtilis (sequences based in crystallographic structure). They were aligned with predicted protein sequences without signal peptides (presumably 25 to 28 amino-terminal peptide), considering only the conserved domains of EXPN. Multiple alignments were analyzed using MegAlign (CLUSTALW) and a phylogenetic tree of EXPN proteins was constructed using Lasergene software. The sunflower genome portal details the Gene Ontology enrichment tests with Blast2GO Pro (one-sided Fisher’s exact tests, false discovery rate of < 0.05) [42]. This information is reported in this study for each putative EXPN gene, together with the gene model and genome localization.

### Principal component analysis, hierarchical clustering, and heatmaps

A heatmap was created to facilitate the graphical interpretation of the relationships between 13 different grain samples (ovary, pericarp and embryo) in different developmental stages using the Clustvis online tool with default settings ([42]; https://biit.cs.ut.ee/clustvis/). Heatmap can be used to visualize the data matrix of EXPN expression; the values in the matrix are color-coded, and we clustered the rows (EXPN expression patterns) by calculating all pairwise distances. Hierarchical clustering was performed using Pearson’s correlation distance [83]. The dimensional expression data was reduced to two dimensions using Principal Component Analysis (PCA). Transformed and normalized gene expression values with log2 were used for analyzing the hierarchical clustering and PCA.

### Statistical analysis

Data of variables and parameters of the ovary and grain growth dynamics were assessed using ANOVA (significant effects at *P* < 0.05 for each factor and interactions), according to the experimental design described above. Regression analyses were used to evaluate the degree of association between variables using Statgraphics Centurion XVI software.

### Correlation analysis

Correlation analysis between phenotypic data and gene expression was performed by R software (version 3.3.3), using the “rsgcc” package with the Gini correlation metric [84]. The *p*-value was calculated with 10,000 permutations with P < 0.05 as the chosen cut-off.

## Additional files

Additional file 1: **Figure S1.** Identity and divergence percentage between EXPN evaluated based on multiple sequence alignment. Sequence aligngments of available full length amino acid sequence with EXPN signal peptide removed. SignalP 3.0 Server software was used to predict the signal peptide cleavage sites. (PDF 39 kb)

Additional file 2: **Figure S2.** Grain dynamics and relative expression patterns of six EXPN during ovary and grain growth in two sunflower genotypes under two source-sink treatments. (PDF 266 kb)

Additional file 3: **Figure S3.** Expression of putative EXPN in various sunflower tissues, according to the transcriptome database (heliagene). A. Scheme of EXPN15 expression. B. Scheme of EXPN4 expression. (RPKM: reads per kilobase per million mapped reads) were chosen. (PDF 187 kb)

Additional file 4: **Table S1.** Characteristics of putative sunflower EXPN. Accession name, chromosome localization, nucleotide and peptide sequence length, Blast2GO, orthologs, and % identity. Data is from the sunflower genome database (Heliagene portal). (PDF 106 kb)

Additional file 5: **Figure S4.** Multiple sequence alignment of predicted protein sequences corresponding to plant orthologs of EXPN7 and eight putative sunflower EXPN. Identical amino acids are shown with black backgrounds, and different amino acids are shown without backgrounds. The potential putative catalytic domain (N-terminal) is indicated by a horizontal blue bar and the putative cellulose binding domain (C-terminal) is indicated by a horizontal red bar; both were predicted using ScanProsite. Multiple alignment was done using MegAlign software. (PDF 285 kb)

## Abbreviations

DFA: days from anthesis
EXPN: expansin
GW: grain weight
