# Supplementary material for: Expansin genes expression in growing ovaries and grains of sunflower are tissue-specific and associate with final grain weight
Source: BMC Plant Biol. 2018 Dec 4;18:327. doi: 10.1186/s12870-018-1535-7 (PMC6280438; doi:10.1186/s12870-018-1535-7)
Supplement: Supplementary file 1 — Figure S1. Identity and divergence percentage between EXPN evaluated based on multiple sequence alignment. Sequence aligngments of available full length amino acid sequence with EXPN signal peptide removed. SignalP 3.0 Server software was used to predict the signal peptide cleavage sites. (PDF 39 kb) [file 12870_2018_1535_MOESM1_ESM.pdf]

Percent Identity

Divergence

	1	2	3	4	5	6	7	8	9	10	11	12	13	14	15	16		
1		90.7	90.7	92.5	85.7	81.7	83.5	72.9	82.1	62.9	71.9	82.1	71.8	74.7	14.9	27.3	1	TRIAE_CS42_5DL_TGACv1_435145_AA1446640
2	9.9		92.5	88.1	85.3	83.0	83.0	72.9	83.5	63.8	71.9	82.1	71.3	73.3	14.9	27.3	2	EXPA6 Brachypodium
3	9.9	7.9		89.8	87.5	87.5	87.1	74.7	87.5	64.7	73.3	87.1	72.2	74.2	14.9	27.3	3	LOC_Os03g60720.1 EXPA RICE
4	7.9	13.0	11.0		84.4	81.7	82.6	71.9	83.0	62.9	70.6	83.5	70.4	72.9	15.8	26.9	4	Alpha-expansin4_Zea mays
5	15.9	16.4	13.7	17.6		90.2	88.8	76.5	91.1	66.2	75.6	89.3	73.6	76.5	16.8	27.8	5	EXPA soybean
6	21.0	19.3	13.7	21.0	10.6		86.6	75.1	95.5	66.2	73.8	95.5	73.1	75.6	16.8	27.3	6	Expa7 sunflower
7	18.7	19.3	14.3	19.9	12.1	14.8		75.6	85.7	65.8	74.7	86.2	73.1	76.0	14.9	26.9	7	EXPA4 Arabidopsis
8	33.7	33.7	31.0	35.1	28.3	30.3	29.6		75.1	68.6	83.7	75.1	80.6	95.9	17.4	26.8	8	Putative EXP1 HaT13l014971
9	20.5	18.7	13.7	19.3	9.5	4.6	15.9	30.3		66.2	73.8	94.6	72.7	75.6	16.3	27.3	9	Putative EXP3 HaT13l005436
10	50.7	49.1	47.4	50.7	44.7	44.7	45.5	40.5	44.7		67.7	65.8	65.6	65.9	16.4	29.4	10	Putative EXP11 HaT13l018566
11	35.1	35.1	33.0	37.3	29.6	32.3	31.0	18.4	32.3	42.1		73.3	88.0	82.4	18.9	29.1	11	Putative EXPA1 HaT13l009552
12	20.5	20.5	14.3	18.7	11.6	4.6	15.3	30.3	5.6	45.5	33.0		72.2	75.6	15.8	27.3	12	Putative EXPA4|HaT13l004607
13	35.4	36.2	34.7	37.7	32.5	33.3	33.3	22.6	34.0	45.9	13.2	34.7		78.7	17.5	28.8	13	Putative EXPA10 Ha412T4l900C0S1
14	31.0	33.0	31.6	33.7	28.3	29.6	28.9	4.2	29.6	45.3	20.2	29.6	25.1		16.9	27.2	14	Putative EXPA15 HaT13l025136
15	332.0	332.0	332.0	311.0	292.0	292.0	332.0	282.0	301.0	299.0	260.0	311.0	282.0	291.0		12.8	15	EXLX1 Bacillus subtilis
16	178.7	178.7	178.7	182.3	175.2	178.7	182.3	183.1	178.7	163.6	165.8	178.7	167.6	179.3	386.0		16	EXPB1 Maize
	1	2	3	4	5	6	7	8	9	10	11	12	13	14	15	16		

Figure S1
