# Supplementary figures and images for: Expansin genes expression in growing ovaries and grains of sunflower are tissue-specific and associate with final grain weight
Source: BMC Plant Biol. 2018 Dec 4;18:327. doi: 10.1186/s12870-018-1535-7 (PMC6280438; doi:10.1186/s12870-018-1535-7)

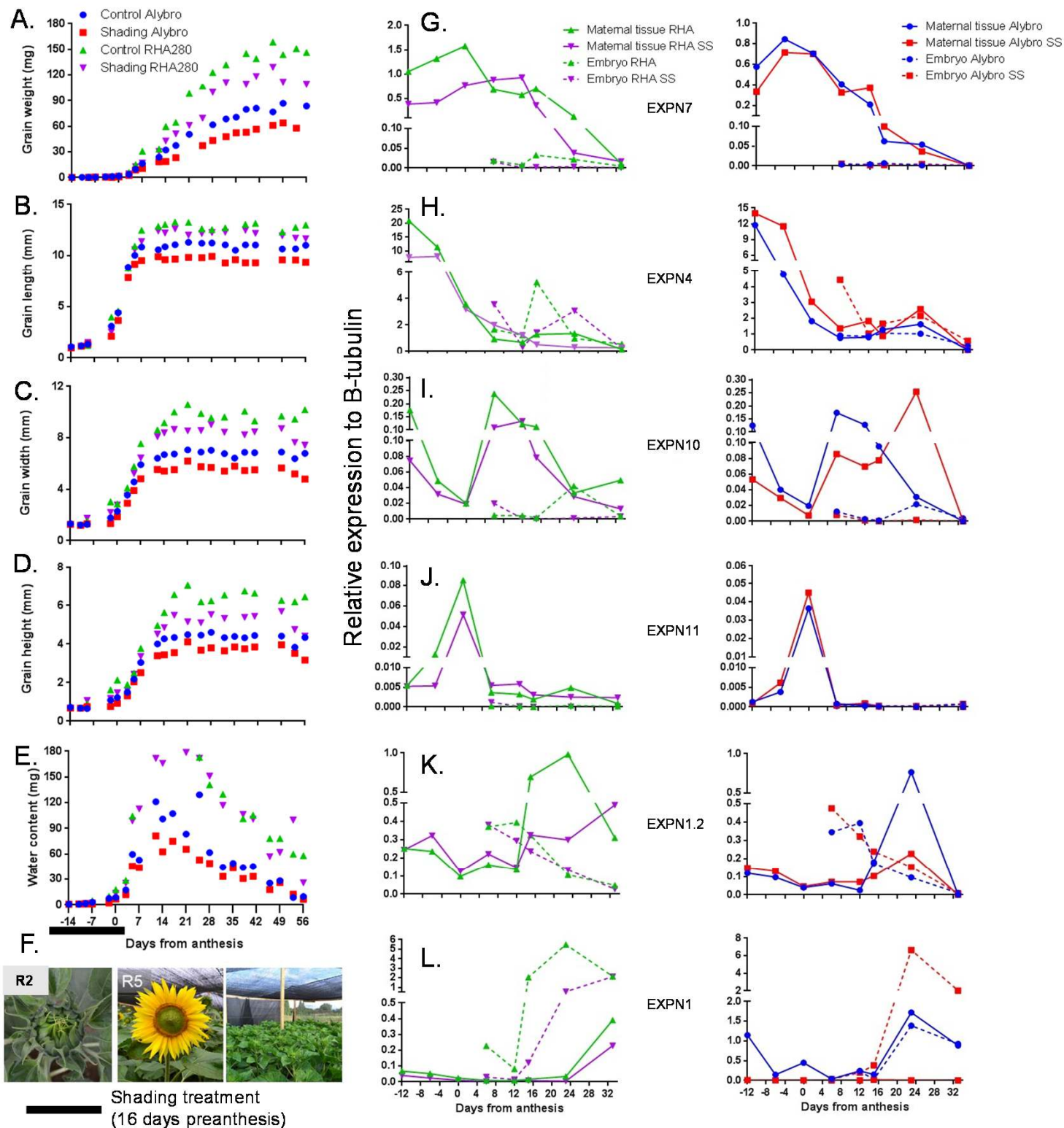

**Figure S2**

Supplement: Supplementary file 2 — Figure S2. Grain dynamics and relative expression patterns of six EXPN during ovary and grain growth in two sunflower genotypes under two source-sink treatments. (PDF 266 kb) [file 12870_2018_1535_MOESM2_ESM.pdf]

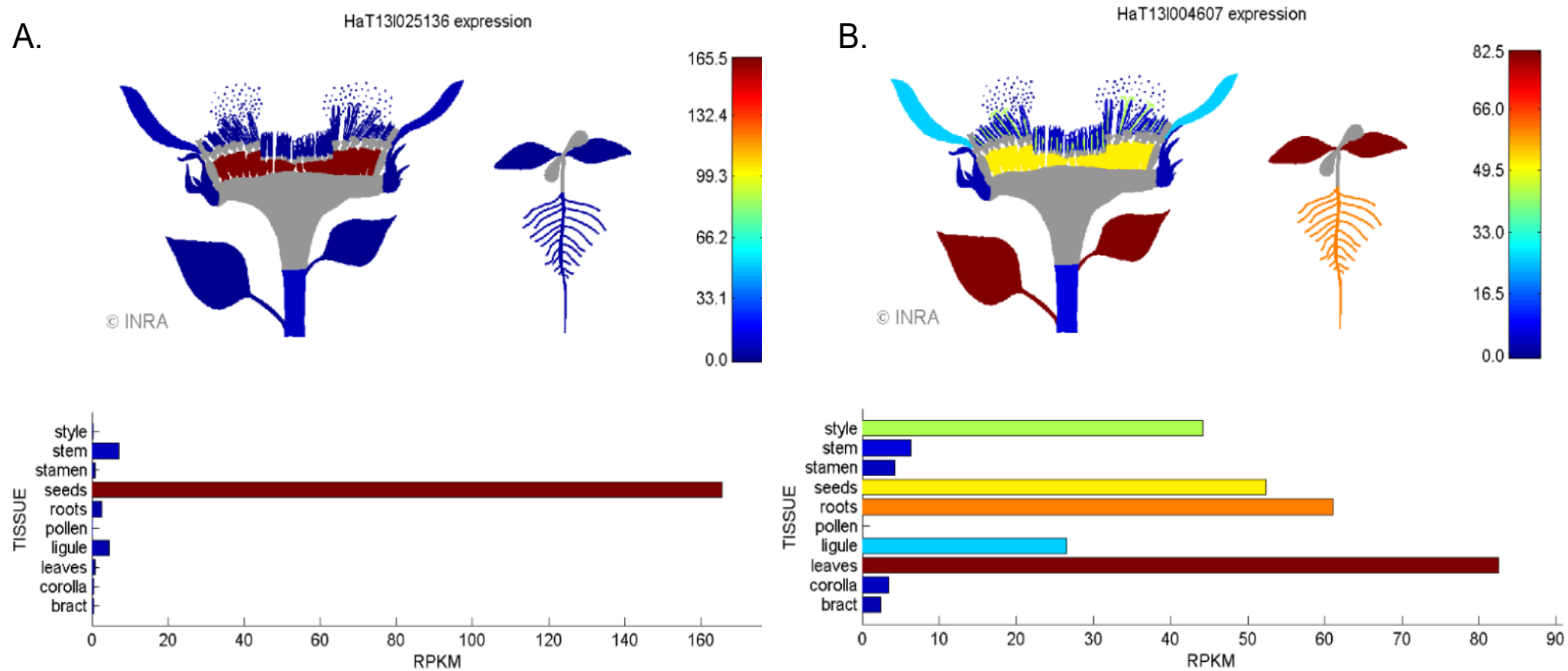

**Figure S3**

Supplement: Supplementary file 3 — Figure S3. Expression of putative EXPN in various sunflower tissues, according to the transcriptome database (heliagene). A. Scheme of EXPN15 expression. B. Scheme of EXPN4 expression. (RPKM: reads per kilobase per million mapped reads) were chosen. (PDF 187 kb) [file 12870_2018_1535_MOESM3_ESM.pdf]

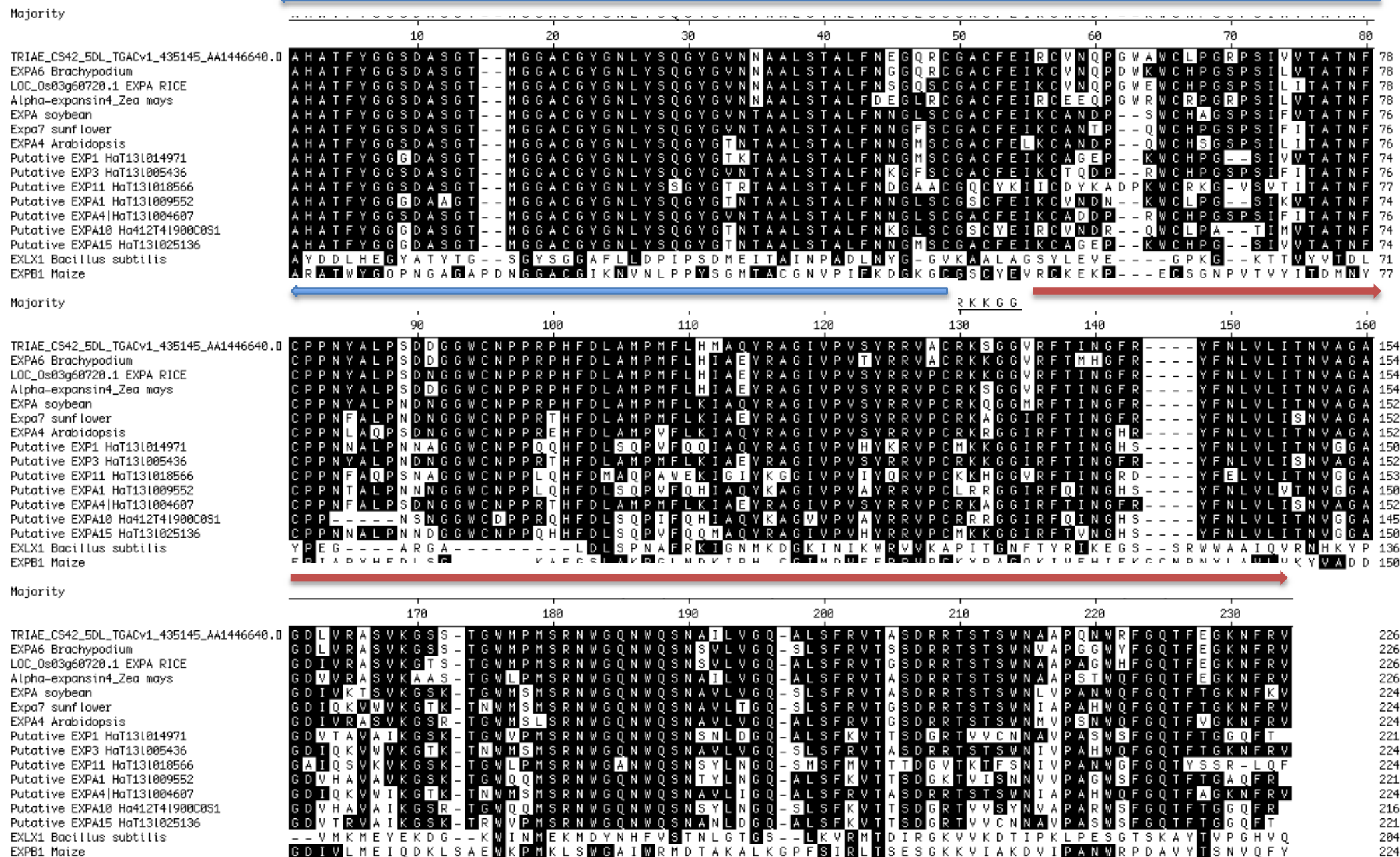

Figure S4

Supplement: Supplementary file 5 — Figure S4. Multiple sequence alignment of predicted protein sequences corresponding to plant orthologs of EXPN7 and eight putative sunflower EXPN. Identical amino acids are shown with black backgrounds, and different amino acids are shown without backgrounds. The potential putative catalytic domain (N-terminal) is indicated by a horizontal blue bar and the putative cellulose binding domain (C-terminal) is indicated by a horizontal red bar; both were predicted using ScanProsite. Multiple alignment was done using MegAlign software. (PDF 285 kb) [file 12870_2018_1535_MOESM5_ESM.pdf]
