# Supplementary material for: Expansin genes expression in growing ovaries and grains of sunflower are tissue-specific and associate with final grain weight
Source: BMC Plant Biol. 2018 Dec 4;18:327. doi: 10.1186/s12870-018-1535-7 (PMC6280438; doi:10.1186/s12870-018-1535-7)
Supplement: Supplementary file 4 — Table S1. Characteristics of putative sunflower EXPN. Accession name, chromosome localization, nucleotide and peptide sequence length, Blast2GO, orthologs, and % identity. Data is from the sunflower genome database (Heliagene portal). (PDF 106 kb) [file 12870_2018_1535_MOESM4_ESM.pdf]

Table S1.

Characteristics	PUTATIVE EXPANSINS							
	**Expansin A4 (HaEXPN4)**	**Expansin A7 (HaEXPN7)**	**Expansin 10 (HaEXPN10)**	**Expansin 11 (HaEXPN11)**	**Expansin 1 (HaEXPN1)**	**Expansin A1 (HaEXPN1.2)**	**Expansin 3 (HaEXPN3)**	**Expansin A15 (HaEXPN15)**
**Accession HaT13I**	HaT13l004607_1_AA	HaT13l007346_1_AA	HaT13l009575_1_AA	HaT13l018566_1_AA	HaT13l014971_1_AA	HaT13l009552_1_AA	HaT13l005436_1_AA	HaT13l025136_1_AA
**Locus Tag (gene) accession XRQ**	HanXRQChr01g0009521	HanXRQChr06g0178721	HanXRQChr08g0210821	HanXRQChr13g0386941	HanXRQChr16g0503121	HanXRQChr17g0570851	HanXRQChr08g0236981	HanXRQChr09g0270611
**Chromosome**	HanXRQChr01	HanXRQChr06	HanXRQChr08	HanXRQChr13	HanXRQChr16	HanXRQChr17	HanXRQChr08	HanXRQChr09
**Start**	64064838	51216670	9589958	2469795	21219190	214553944	151037735	189762140
**End**	64068076	51219551	9592439	2471756	21222064	214555988	151039716	189763612
**Strand**	(- strand)	(- strand)	(+ strand)	(- strand)	(- strand)	(- strand)	(- strand)	(+ strand)
**ORF Length (bp)**	3239	2882	2482	1962	2875	2045	1982	1473
**Protein sequence length(aa)**	257	273	258	254	274	311	257	265
**Blast2GO**	Cell Wall, membrane, plant-type cell wall organization, extracellular region	Cell Wall, membrane, plant-type cell wall organization, extracellular region	Cell Wall, membrane, plant-type cell wall organization, extracellular region	Cell Wall, membrane, plant-type cell wall organization, extracellular region	Cell Wall, membrane, plant-type cell wall organization, extracellular region	Cell Wall, membrane, plant-type cell wall organization, extracellular region	Cell Wall, membrane, plant-type cell wall organization, extracellular region	Cell Wall, membrane, plant-type cell wall organization, extracellular region
Orthologous Arabidopsis	AT2G39700.1	AT2G39700.1	AT1G26770.2	AT1G20190.1	AT1G69530.3	AT1G69530.2	AT2G37640.1	AT2G03090.1
**Identity**	80.54%	82.49%	76.40%	75.21%	80.00%	83.13%	80.93%	79.18%
***Orthologous* Soybean**	Glyma.12G062700.1	Glyma.02G248500.1	Glyma.01G050100.1	Glyma.17G260400.1	Glyma.17G101400.4	Glyma.01G050100.1	Glyma.02G248500.1	Glyma.04G167800.1
**Identity**	84.23%	86.85%	81.20%	76.86%	83.70%	86.61%	85.77%	78.73%
***Orthologous* B. dystachyon**	Bradi1g03640.1	Bradi1g03640.1	Bradi2g22290.1	Bradi5g04120.1	Bradi1g61190.1	Bradi2g22290.1	Bradi1g03640.1	Bradi2g53580.1
**Identity**	79.34%	81.82%	72.13%	69.08%	70.24%	75.82%	80.33%	76.09%
